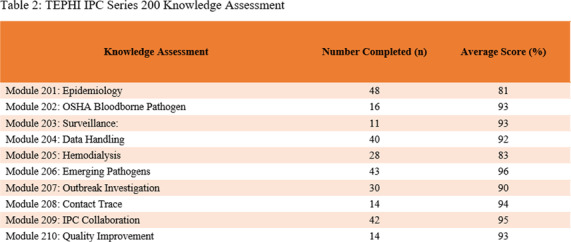# Development, Delivery, and Evaluation of the Texas Epidemic Public Health Institute (TEPHI) Infection Control Lecture 200 Series

**DOI:** 10.1017/ash.2025.343

**Published:** 2025-09-24

**Authors:** Kayla Ruch, Anabel Rodriguez, Janelle Rios Janelle Rios

**Affiliations:** 1 The University of Texas Health Science Center and Texas Epidemic Public Health Institute (TEPHI)

## Abstract

**Background:** The Texas Epidemic Public Health Institute (TEPHI) aims to safeguard public health and bolster the economy by preparing for infectious disease outbreaks. The Infection Prevention and Control Webinar (IPC) 200 series of the Small Rural Healthcare Preparedness offers free educational resources and continuing education for public health and healthcare personnel responsible for infection prevention programs across ten lectures from requested topics from TEPHIs IPC 100 series. **Methods:** Data from the second year of the Infection Prevention and Control lecture series were collected using attendee registration and attendance data, knowledge assessments, and post-lecture evaluation surveys via WebEx®, QuestionPro®, and Microsoft Teams®. The modules were developed using resources from the Association for Professionals in Infection Control and Epidemiology (APIC), the Occupational Safety and Health Administration (OSHA), the Centers for Disease Control and Prevention (CDC), The Joint Commission (TJC), and Centers for Medicare and Medicaid Services. **Results:** The series had 1,088 attendees to the live lectures and generated 3,103 YouTube views. Lectures were accredited for 1.0 hours of public health education and a-IPC certification, with 8 of 10 sessions offering 1.0 continuing education hours for CIC certifications for infection preventionists. Of the 286 participants completing knowledge assessments, the average score was 91% (range: 81% in Module 201 to 96% in Module 206). Post-evaluations (n=280) rated the content highly (mean: 4.83/5) for beneficial, easy to understand, and clear/concise. Additionally, 90.4% of respondents indicated plans to implement the knowledge gained, and 98.9% expressed interest in attending future sessions. **Conclusion:** The Infection Control lecture series improved participants’ knowledge of infection prevention and control best practices. By disseminating evidence-based education and providing no-cost continuing education, the series equips healthcare personnel with the tools to foster safer environments for patients and staff in healthcare settings.

**Figure:**
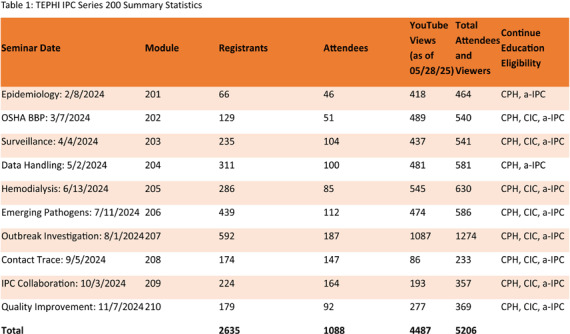

**Figure:**